# Virology Journal Reviewer Acknowledgement 2015

**DOI:** 10.1186/s12985-016-0484-8

**Published:** 2016-03-07

**Authors:** Linfa Wang

**Affiliations:** Program in Emerging Infectious Diseases, Duke-NUS Graduate Medical School, Singapore, Singapore

## Abstract

The editors of *Virology Journal* would like to thank all our reviewers who have contributed to the journal in Volume 12 (2015). The success of any scientific journal depends on an effective and strict peer review process and *Virology Journal* could not operate without your contribution.

Our record time from submission to online, open access, publication in 2015 was 36 days for a Research Article (Virol J 12:107, 2015). This is a great achievement by any standard. We look forward to your continuous support of *Virology Journal* either as an invited reviewer or a contributing author in the years to come.

**Mohamed Abdel-Rahman**

Egypt

**Laith Abu-Raddad**

Qatar

**Rosita Accardi**

France

**Evelien Adriaenssens**

South Africa

**Muhammad Afzal**

Pakistan

**Anurodh Agrawal**

USA

**Bashir Ahmad**

Pakistan

**Christopher Aiken**

USA

**Alí Alejo**

Spain

**Nezar Al-hebshi**

Saudi Arabia

**M Ali**

Pakistan

**Jean-Pierre Allain**

UK

**Gabriel Almeida**

Brazil

**Mohammed Alsharifi**

Australia

**Jaffar Al-Tawfiq**

Saudi Arabia

**Flávio Amaral**

Brazil

**Rebecca Ambrose**

Australia

**Danielle Anderson**

USA

**Alessandro Antonelli**

Italy

**Tanya Applegate**

Australia

**Armando Arias**

UK

**Minetaro Arita**

Japan

**Sassan Asgari**

Australia

**Andrea Astolfi**

Italy

**Claire Atkinson**

UK

**Ajax M. Atta**

USA

**Walid Azab**

Germany

**Hafsa Aziz**

Pakistan

**Eduardo Azziz-Baumgartner**

USA

**Kasia Bachanek-Bankowska**

UK

**Inés Badano**

Argentina

**Dalan Bailey**

UK

**Udeni Balasuriya**

USA

**Alejandro Banda**

USA

**Bruce Banfield**

Canada

**Ashley Banyard**

UK

**Khaled Barakat**

Canada

**John Barr**

UK

**Alan Barrett**

USA

**Naina Barretto**

USA

**Erik Barton**

USA

**Lorena Bavia**

Brazil

**David Beasley**

USA

**Maria Serena Beato**

Italy

**Simon Beddows**

UK

**Andrew Bell**

UK

**Brendan Bell**

Canada

**Mikael Berg**

Sweden

**Analia Berinstein**

USA

**Jesus F Bermejo-Martin**

Spain

**Sonja Best**

USA

**Preeti Bharaj**

India

**Bishnupriya Bhattacharya**

UK

**Jayanta Bhattacharya**

India

**Kelly Alves Bicalho**

USA

**Esther Blanco**

Spain

**Sandra Blome**

Germany

**Rogier Bodewes**

Netherlands

**Silvia Bofill-Mas**

Spain

**Stephen Bond**

USA

**Cláudio Bonjardim**

Brazil

**Shubhada Bopegamage**

Slovakia

**Juliano Bordignon**

Brazil

**Silvia Boscardin**

Brazil

**Albert Bosch**

Spain

**Emanuela Bostjancic**

Slovenia

**Viviane Fongaro Botosso**

USA

**Crystal Boudreaux**

USA

**Carla Braconi**

Brazil

**Paulo Brandao**

Brazil

**Curtis Brandt**

USA

**Kelly Brayton**

USA

**Amélie Brebion**

France

**Andrew Broadbent**

USA

**Ivan Brukner**

Canada

**Wolfram Brune**

Germany

**Celina Buscaglia**

Argentina

**Ismael Bustos**

Mexico

**Jeff Butler**

Australia

**Mathias Buttner**

Germany

**Raul Cachau**

USA

**Sarah Caddy**

UK

**Tina Cairns**

USA

**Amanda Calvert**

USA

**Marco Antônio Campos**

Brazil

**Patricia Cane**

UK

**Chunxia Cao**

USA

**Patrizia Caposio**

USA

**Guadalupe Carballal**

Argentina

**Jill Carr**

Australia

**James Carroll**

USA

**José M. Casasnovas**

Spain

**Elisabetta Caselli**

Italy

**John Casey**

USA

**Bryce Chackerian**

USA

**Jasper Fuk Woo Chan**

Hong Kong

**Jinhong Chang**

USA

**Wen Chang**

USA

**Remi Charrel**

France

**Ranjit Chauhan**

USA

**Hualan Chen**

China

**Weiqiang Chen**

Australia

**Zhongbin Chen**

China

**Han Cheng**

USA

**Wan Shoo Cheong**

Malaysia

**Hui-Ling Chiou**

Taiwan

**Ashok Chockalingam**

USA

**Kyoung-Seong Choi**

Korea, South

**Chungwon Chung**

USA

**Young-Hwa Chung**

Korea, South

**Rollie Clem**

USA

**Jan Clement**

Belgium

**Gary Clifford**

France

**Christine Clouser**

USA

**Karen Coats**

USA

**Don Coen**

USA

**Aidan Coffey**

Ireland

**Randall Cohrs**

USA

**Samuel Cordey**

Switzerland

**Victor Corman**

Germany

**Louise Cosby**

UK

**Andre Luis Costa-da-Silva**

Brazil

**Maria Isabel Costafreda**

USA

**Susan Cotmore**

USA

**Lauren Cowley**

UK

**Rebecca Creamer**

USA

**Robert Cross**

USA

**Jie Cui**

USA

**Lunbiao Cui**

China

**Cristina Cunha**

USA

**Jil Czarnecki**

USA

**Markus Czub**

Canada

**Ajmal Dalwai**

Kuwait

**Jose-Antonio Daros**

Spain

**Javid Dastia**

Pakistan

**David Davido**

USA

**Andrew Davison**

UK

**Erick De la Cruz-Hernandez**

Mexico

**Danilo de Oliveira**

Brazil

**R. L. de Swart**

USA

**Emmie de Wit**

USA

**Yannick Debing**

Belgium

**Nicola Decaro**

Italy

**Jose Del Campo**

Spain

**Edson Delatorre**

Brazil

**Henry Delecluse**

Germany

**Francis Delpeyroux**

France

**Neal DeLuca**

USA

**Eric Delwart**

USA

**Xufang Deng**

USA

**Yi-Mo Deng**

Australia

**Paul Deny**

France

**Philippe Desprès**

France

**J.A. Díaz-Pendon**

Spain

**Diego Diel**

USA

**Ralf Dietzgen**

Australia

**Ronald Dijkman**

Switzerland

**Biao Ding**

USA

**Chan Ding**

China

**Virginie Doceul**

France

**Katarzyna Domanska-Blicharz**

Poland

**Robert Domaoal**

USA

**Chunsheng Dong**

China

**Ming Duan**

China

**Stephen Dunham**

UK

**Bas Dutilh**

Netherlands

**Jose Echenique**

Argentina

**John-Sebastian Eden**

Australia

**Bernhard Ehlers**

Germany

**Maryna Eichelberger**

USA

**Anna Eis-Huebinger**

Germany

**Johanna Ekstrom**

Sweden

**Joakim Esbjörnsson**

Sweden

**Fernando Escriu**

Spain

**Jennifer Evans**

UK

**Catherine Ewen**

USA

**Thomas Fabrizio**

USA

**Shufang Fan**

USA

**Zaifeng Fan**

China

**Minggang Fang**

USA

**Rolf Fendel**

Germany

**Zongdi Feng**

USA

**Fernando Ferreira**

Portugal

**Burtram Fielding**

South Africa

**James Fielding**

Australia

**J Fokom-Domgue**

Cambodia

**Allex Fonseca**

Brazil

**Craig Forrest**

USA

**Patrick Forterre**

France

**Lee Fortunato**

USA

**Jeffrey Foster**

USA

**Arthur Frampton**

USA

**Alexander Freiberg**

USA

**Antonio Carlos de Freitas**

Brazil

**Lisa Frenkel**

USA

**Matthew Frieman**

USA

**Ilya Frolov**

USA

**Shuetsu Fukushi**

Japan

**Overta Fuller**

USA

**Gulsah Gabriel**

Germany

**Carl A. Gagnon**

Canada

**Inmaculada Galindo**

Spain

**Llilianne Ganges**

Spain

**Michael Gantier**

Australia

**Feng-Shan Gao**

China

**Yulong Gao**

China

**Yuwei Gao**

USA

**Marisa Gariglio**

Italy

**Nina Milutin Gasperov**

USA

**Hiroyuki Gatanaga**

Japan

**Phil Gauger**

USA

**Danielle Gava**

Brazil

**Edward Gershburg**

USA

**Reena Ghildyal**

Australia

**Shin-je Ghim**

USA

**Abdeljelil Ghram**

Tunisia

**Kathleen Gibson**

USA

**Ivone Giffard-Mena**

Mexico

**Leona Gilbert**

Finland

**Daniel Gomez**

Brazil

**Radhika Gopal**

USA

**Brian Gowen**

USA

**Simon Graham**

UK

**Michael Grant**

Canada

**Urs Greber**

Switzerland

**Charles Grose**

USA

**Rodrigo Guabiraba**

France

**Larisa Gubareva**

USA

**Susana Guix**

Spain

**Fei Guo**

China

**JQ Guo**

USA

**Ju-Tao Guo**

USA

**Nimesh Gupta**

France

**Phalguni Gupta**

USA

**Kurt E Gustin**

USA

**Andy Haegeman**

Belgium

**Chenggui Han**

China

**Tomas Hanke**

UK

**Steve Hanson**

USA

**Jennifer L. Harcourt**

USA

**Isabel Haro**

Spain

**Robert Harrison**

USA

**Stephen Hartung**

USA

**Heli Harvala**

UK

**Ben Hause**

USA

**Zifu He**

China

**Alireza Heidari**

Italy

**Jan Heidemann**

Germany

**Karla Helbig**

Australia

**Ekaterina Heldwein**

USA

**Michael Hess**

Austria

**Masayuki Horie**

Japan

**Zhongjie Hu**

China

**Cheng Huang**

USA

**Li-Rung Huang**

Taiwan

**Wenya Huang**

Taiwan

**Yhu-Chering Huang**

Taiwan

**Yu Huang**

China

**Zhong Huang**

China

**Linda Hueston**

Australia

**Hee Jae Huh**

Korea, South

**Amy Hulme**

USA

**Liang-Yi Hung**

Taiwan

**Aeron Hurt**

Australia

**Gisela Hussey**

USA

**Lih-Hwa Hwang**

Taiwan

**Raquel Ibáñez**

Spain

**Sabarish Indran**

India

**Aaron Irving**

Australia

**Masanori Isogawa**

Japan

**Koji Itahana**

Singapore

**Yoshihiro Izumiya**

USA

**Mark W. Jackwood**

USA

**Bert Jacobs**

USA

**Graeme Jacobs**

South Africa

**Keith Jarosinski**

USA

**Lorena Jemersic**

Croatia

**Shijin Jiang**

China

**Yanfang Jiang**

China

**Peirong Jiao**

China

**Qi Jin**

China

**Emma Job**

Belgium

**Eric Johannsen**

USA

**Clinton Jones**

USA

**Patricia Jorquera**

USA

**Toms Joseph**

USA

**Sandra Junglen**

Germany

**Tsutomu Kageyama**

Japan

**Manjula Kalia**

India

**Tatsuo Kanda**

Japan

**Beatrix Kapusinszky**

USA

**P. Karanis**

Germany

**Takanobu Kato**

Japan

**Barb Katzenback**

USA

**Benedikt Kaufer**

Germany

**Yoshiiku Kawakami**

Japan

**Ghazi Kayali**

USA

**Matthew Kaye**

Australia

**Yang Ke**

China

**Tuija Kekarainen**

Spain

**Alyson Kelvin**

Canada

**Shannon Kenney**

USA

**Recep Kesli**

USA

**Hossam Khamis**

Egypt

**Mohsin Khan**

USA

**Zafar Khan**

USA

**Sunil Khattar**

USA

**Frederick Kibenge**

Canada

**Chul-Joong Kim**

Korea, South

**Shin-Hee Kim**

USA

**Yunjeong Kim**

USA

**Christine King**

USA

**Matt Kitson**

Australia

**Stephanie Kliethermes**

USA

**T. Klimkait**

Switzerland

**Jonas Klingstrom**

Sweden

**William Klitz**

USA

**Bernard Klonjkowski**

France

**Barbara Klupp**

Germany

**Jeffrey Koehler**

USA

**Andrea Koishi**

Brazil

**Tuckweng Kok**

Australia

**Ganesh Kondabattula**

India

**Misa Korva**

Slovenia

**Konstantin Kousoulas**

USA

**Manoj Krishnan**

Singapore

**David Kristensen**

USA

**Diogo Kuczera**

Brazil

**Sachin Kumar**

India

**Marc Kvansakul**

Australia

**Cristiano Lacorte**

Brazil

**Ole Lagatie**

Belgium

**Luis Lamberti Da Silva**

Brazil

**Tatiana Lanzieri**

USA

**Susanna Lau**

Hong Kong

**Chang-Won Lee**

USA

**David Lefebvre**

Belgium

**Jolie Leonard**

USA

**Amel Letaief**

Tunisia

**Avram Levy**

Australia

**Arnaud L'Huillier**

Switzerland

**Chengjun Li**

China

**Fang Li**

USA

**Guangxing Li**

China

**Kang Sheng Li**

China

**Linlin Li**

USA

**Qingxue Li**

USA

**Sean Li**

Canada

**Zejun Li**

China

**Zhenghe Li**

China

**Chen Liang**

Canada

**Mifang Liang**

China

**Min Liao**

China

**Honghui Lin**

China

**Junyan Lin Lin**

USA

**Kai-Shu Ling**

USA

**Martin Linster**

Singapore

**Howard Lipton**

USA

**Andrea Lisotti**

Italy

**Chia-Chyi Liu**

Taiwan

**Helene Minyi Liu**

Taiwan

**Hongmei Liu**

China

**Qinghui Liu**

USA

**Sarah Elisabeth Lloyd**

UK

**Thomas London**

USA

**Sarah Londrigan**

Australia

**C Lopez-Galindez**

Spain

**Franco Lucchini**

Italy

**Juan Ludert**

Mexico

**Juan Ernesto Ludert**

Mexico

**Yan Long Edmund Lui**

Australia

**Alexander Lukashev**

Russia

**Mang Ma**

Canada

**Wei Ma**

China

**Yijie Ma**

USA

**M Macdonald**

USA

**Jason Mackenzie**

Australia

**Tariq Madani**

Saudi Arabia

**Ole Madsen**

Netherlands

**Suresh Mahalingam**

Australia

**Kapil Maithal**

USA

**Eugene Major**

USA

**Kristiina Mäkinen**

Finland

**Imran Malik**

Pakistan

**Niwat Maneekarn**

Thailand

**Balaji Manicassamy**

USA

**Gayathri Manokaran**

Australia

**Shahid Mansoor**

Pakistan

**Darren Martin**

South Africa

**Miguel Angel Martinez**

Spain

**Encarna Martinez Salas**

Spain

**James Maruniak**

USA

**Hugh Mason**

USA

**William Mason**

USA

**Keiichiro Matsukura**

Japan

**Budhaditya Mazumdar**

USA

**Alison McBride**

USA

**John Mcbride**

Australia

**Dennis McCance**

USA

**Anita McElroy**

USA

**Alistair McGrgor**

USA

**Colin McInnes**

USA

**Clive McKimmie**

UK

**Sean Meaden**

UK

**Adam Meijer**

Netherlands

**José A. Melero**

Spain

**Fernando Melo**

Brazil

**Aristobolo Mendes Silva**

Brazil

**Andres Merits**

Estonia

**Scott Michael**

USA

**JM Middeldorp**

Netherlands

**Santiago Mirazo**

USA

**Chad Mire**

USA

**Nischay Mishra**

USA

**Takayuki Miura**

France

**Katsumi Mizuta**

Japan

**Ugo Moens**

Norway

**Mahmoud Mohey Elhaig**

Egypt

**Debasis Mondal**

USA

**Mario Mondelli**

Italy

**Cary Moody**

USA

**Marcelo Moret**

Brazil

**Kouichi Morita**

Japan

**Junona Moroianu**

USA

**Adi Moseri**

Israel

**John Moss**

USA

**Bruno Mota**

Brazil

**Patricia Mulrooney-Cousins**

Canada

**Primitivo Murillo**

Spain

**Eain Murphy**

USA

**Noriyo Nagata**

Japan

**Tatsuya Nagata**

Brazil

**Eva Nagy**

Canada

**Rayapati Naidu**

USA

**Keisuke Nakagawa**

Japan

**S Nakamoto**

Japan

**Farooq Nasar**

USA

**Chris Netherton**

UK

**Lisa F.P. Ng**

Singapore

**Terry Fei Fan Ng**

USA

**Tsai Nga Wing Polly**

Hong Kong

**John Nicholls**

Hong Kong

**Xianzhou Nie**

Canada

**Masa Niikura**

USA

**Veljko Nikolin**

Germany

**Andreas Nitsche**

Germany

**Jean Jacques Noubiap**

South Africa

**Antoine Nougairede**

France

**Martin M Nyaga**

USA

**Mylène Ogliastro**

France

**Tomoichiro Oka**

USA

**Chioma Okeoma**

USA

**Gene Olinger**

USA

**Hugo Oliveira**

Portugal

**Stefan Oliver**

USA

**Andrea Olmstead**

Canada

**O Opaleye**

Nigeria

**Senthilkumar Palaniyandi**

USA

**Anandan Paldurai**

USA

**Giorgio Palu**

Italy

**Ana Luiza Pamplona Mosimann**

Brazil

**Hanu Pappu**

USA

**Prasad Paradkar**

Australia

**Edyta Paradowska**

Poland

**Rebecca Parr**

USA

**Colin Parrish**

USA

**Mehul Patel**

India

**Kunj Pathak**

USA

**Marco Patrone**

Italy

**Bramhadev Pattnaik**

India

**Alex Pauvolid-Correa**

Brazil

**Andrea Peralta**

Argentina

**Luciane Maria Perazzolo**

Brazil

**Allan Peres-da-Silva**

Brazil

**Eva Perez-Martin**

UK

**Jody Peters**

Australia

**My VT Phan**

USA

**Aguinaldo Pinto**

Brazil

**Mauro Pistello**

Italy

**Leo Poon**

Hong Kong

**Welkin Pope**

USA

**Mohamad Amin Pourhoseingholi**

USA

**Hamid Pourianfar**

Iran

**Ramesh Prabhu**

USA

**Tatiana Prado**

Brazil

**Natalie Prow**

Australia

**Krzysztof Pyrc**

Poland

**Yajuan Qian**

China

**Zhikang Qian**

China

**Chuanling Qiao**

China

**Ling Qing**

China

**Yu Qiu**

Belgium

**Marek Radkowski**

Poland

**Shane Raidal**

Australia

**Daniela Rajao**

USA

**Yogendra Rajawat**

USA

**Simon Rayner**

China

**G Sullivan Read**

USA

**Thipparthi R Reddy**

USA

**Roger Reeves**

USA

**Lili Ren**

China

**Tristan Renault**

France

**Chantal Reusken**

Netherlands

**Bergmann Morais Ribeiro**

Brazil

**Caroline Rigotto**

Brazil

**Bert Rima**

UK

**Espen Rimstad**

Norway

**Ana Rivas-Estilla**

Mexico

**Ashley Roberts**

UK

**Sally Roberts**

UK

**Rosemary Rochford**

USA

**Dolores Rodriguez**

Spain

**Julio Rodriguez**

Australia

**Ann Roman**

USA

**Angela Römer-Oberdörfer**

Germany

**Joan Rose**

USA

**Helene Rosenberg**

USA

**CC Rossetto**

USA

**Barry Rouse**

USA

**Rejane Rua**

USA

**Penny Rudd**

Australia

**Ali Sabahi**

USA

**Ivan Sadowski**

Canada

**Muhammad Saeed**

Pakistan

**Tani Sagna**

Burkina Faso

**Dipongkor Saha**

USA

**Zeinab Said**

USA

**Masayuki Saijo**

Japan

**Bruno Sainz**

Spain

**Mayuko Saito**

Japan

**Margarita Sáiz**

Spain

**Masahiro Sakai**

Japan

**Shamim Saleha**

Pakistan

**Sara Salinas**

France

**Siba Samal**

USA

**Teruo Sano**

Japan

**Flavia Santos**

Brazil

**Daniel Santos Mansur**

Brazil

**Steven Sarrazin**

Belgium

**Carita Savolainen-Kopra**

Finland

**Mark Schleiss**

USA

**Connie Schmaljohn**

USA

**Scott Schmid**

USA

**Paul Schnitzler**

USA

**Jörg Schubert**

Germany

**Amy Schuh**

USA

**Stefan Schwartz**

Sweden

**Matthias Schweizer**

Switzerland

**October Sessions**

USA

**Noemi Sevilla**

Spain

**Tongling Shan**

USA

**Shaobin Shang**

China

**Colin Sharp**

UK

**Huigang Shen**

China

**Masako Shimamura**

USA

**Maya Shmulevitz**

Canada

**Kirsty Short**

Australia

**Deepak Shukla**

USA

**Laura Sichero**

Brazil

**Steven Sijmons**

Belgium

**Guilherme Silveira**

Brazil

**Peter Simmonds**

UK

**Carmen Simón**

Spain

**Mike Skinner**

UK

**Richard Sloan**

UK

**Duncan Smith**

Thailand

**Ina Smith**

Australia

**Teemu Smura**

USA

**M.A. Soares**

Brazil

**Marzia Sohler**

Brazil

**George Sourvinos**

USA

**David Speers**

Australia

**Eduardo Spencer**

Chile

**Fernando Spilki**

Brazil

**V.A. Srinivasan**

India

**Giorgio Stanta**

Italy

**Richard Stanton**

UK

**Phoebe Stewart**

USA

**Norbert Stockhofe-Zurwieden**

Netherlands

**Patricia Stoco**

Brazil

**Stephen Stray**

USA

**Rebecca Strong**

UK

**Daisy M. Strottmann**

Brazil

**Frank Stubenrauch**

Germany

**Mireille St-Vincent**

Canada

**Ih-Jen Su**

Taiwan

**Yvonne Su**

Singapore

**Kazushi Sugimoto**

Japan

**Surong Sun**

China

**Garry Sunter**

USA

**Camille Sureau**

France

**Nobuhiro Suzuki**

Japan

**Sathyamangalam Swaminathan**

India

**Gulam Syed**

USA

**Moriah Szpara**

USA

**Takako Tabata**

USA

**Muhammad Tahir**

Pakistan

**Akinobu Takaki**

Japan

**Emi Takashita**

Japan

**Uehara-Ichiki Tamaki**

Japan

**Catherine Tamalet**

France

**Yasuhito Tanaka**

Japan

**Qiyi Tang**

USA

**Xianchun Tang**

USA

**Mi-Hua Tao**

Taiwan

**Xiaorong Tao**

China

**Alexander Tarr**

UK

**Adam Taylor**

Australia

**Harry Taylor**

USA

**John Taylor**

USA

**Matthew Taylor**

USA

**Maureen Taylor**

South Africa

**Shannon Taylor**

USA

**Peter Tijssen**

Canada

**Eve Todesco**

France

**Conny Tolf**

Sweden

**Dewen Tong**

China

**Alfonsina Trento**

Spain

**Mirko Trillig**

USA

**Srikanth Tripathy**

India

**Ryan Troyer**

USA

**Chien-Te Kent Tseng**

USA

**Tamás Tuboly**

Hungary

**Matthew Turnbull**

USA

**Tobias Tuthill**

UK

**Sukathida Ubol**

Thailand

**Susan Uprichard**

USA

**Antti Vaheri**

Finland

**Antonio Valentin**

USA

**Steven Van Borm**

Belgium

**Tonja van der Kuyl**

Netherlands

**Rogier Van Doorn**

Viet Nam

**Mark van Raaij**

Spain

**Gilberto Vaughan**

USA

**Eliane Veiga da Costa**

Brazil

**Michael Veit**

Germany

**Paola Venier**

Italy

**G Venkatesan**

India

**Vivek Verma**

Korea, South

**Dhanasekaran Vijaykrishna**

Singapore

**Stefan Vilcek**

Slovakia

**Livia Villar**

Brazil

**Veronika Von Messling**

Germany

**Ann Vossen**

Netherlands

**Frank Vreede**

UK

**Domagoj Vucic**

USA

**Per Wallgren**

Sweden

**Hongquan Wan**

USA

**Xiu-Feng (Henry) Wan**

USA

**David Wang**

USA

**Fu-Sheng Wang**

USA

**Jianning Wang**

Australia

**Jianwei Wang**

China

**Xifeng Wang**

China

**Xinping Wang**

China

**Zhiliang Wang**

China

**Brian Ward**

USA

**Ali Waryah**

Pakistan

**Len Wasiloski**

USA

**Shumpei Watanabe**

Japan

**Koichi Watashi**

Japan

**Keith Watson**

Australia

**Roger Watson**

UK

**Elizabeth White**

USA

**Paul Wichgers Schreur**

Netherlands

**Matt Wiebe**

USA

**Brian Wigdahl**

USA

**Susan Williams**

USA

**Trevor Williams**

Mexico

**Joyce Wilson**

Canada

**Frank Wong**

Australia

**Patrick Chiu Yat Woo**

Hong Kong

**Stefan Worgall**

USA

**Grzegorz Jan Wozniakowski**

Poland

**Jianguo Wu**

China

**Steve Wylie**

Australia

**Zhiyong Xi**

USA

**Qingyou Xia**

China

**Yu Xiang**

Cameroon

**Gengfu Xiao**

China

**Xiao-Guang Dou**

USA

**Li Xie**

China

**Yan Xie**

China

**Aiqiang Xu**

China

**Huimin Xu**

Canada

**Kai Xu**

USA

**Wei Xu**

USA

**Teruo Yamashita**

Japan

**Vladimir Yamshchikov**

USA

**Hung-Chih Yang**

Taiwan

**Kui Yang**

USA

**Song Yang**

USA

**Zengqi Yang**

China

**Tahir Yaqub**

USA

**Jianqiang Ye**

China

**Tomoki Yoshikawa**

Japan

**Bin Yu**

China

**Di Yu**

Australia

**Ming-Lung Yu**

Taiwan

**Yuchen Fan**

China

**Zeng Yue**

China

**Tanaka Yuetsu**

Japan

**Nadezhda Yun**

USA

**Ali Zaid**

Australia

**Carlos Rodrigo Zarate-Bládes**

Brazil

**Lingbing Zeng**

China

**Murillo Zerbini**

Brazil

**Bo Zhang**

China

**Dabing Zhang**

China

**Hengmu Zhang**

China

**Yan Zhang**

USA

**Yongliang Zhang**

China

**Guozhong Zhang**

China

**Zhenhua Zhang**

China

**Xiao-Qun Zheng**

China

**Bin Zhou**

USA

**Bin Zhou**

China

**En-Min Zhou**

China

**Hongbo Zhou**

China

**Peng Zhou**

Singapore

**Feng-cai Zhu**

China

**Qiyun Zhu**

China

**Véronique Ziegler-Graff**

France

**Jan Franciszek Zmudzinski**

Poland
